# Investigation on the application of digital guide templates guided dental implantation in China

**DOI:** 10.1186/s12903-023-02750-4

**Published:** 2023-01-22

**Authors:** Yunli Chen, Baohui Su

**Affiliations:** College of Biomedical Engineering, Sichuan University, Chengdu, 610065 Sichuan Province China

**Keywords:** Guided surgery, Dental implant, Accuracy, Application, Survey

## Abstract

**Background:**

The aim of this survey is to investigate the application of digital guide templates (DGTs) across China, and the views and attitudes of oral health professionals toward them.

**Methods:**

This survey was prepared, distributed, and collected by WJX. Chinese oral health professionals were invited to participate in it. The basic information of respondents, the application of DGTs, and the views and attitudes toward their status quo and development were statistically described. Chi-square test was used to evaluate the correlation between the basic information of respondents and the application of DGTs as well as the views and attitudes toward them.

**Results:**

A total of 276 questionnaires were collected, of which 273 were identified as valid. 269 (98.5%) respondents were dental clinical workers, 204 (74.7%) were dental clinical implant workers, and 152 (55.7%) had been engaged in the implant industry for more than five years. The chi-square test showed that working years were significantly correlated with the half-guided, tooth-supported, and mucosa-supported DGTs (*P* < 0.05); and professional backgrounds and working years presented significant differences in the views and attitudes toward the status quo and development of DGTs (*P* < 0.05). The questionnaires also made a preliminary investigation and evaluation on the factors influencing accuracy, indications, doctors’ recommendations and relevant training.

**Conclusion:**

Most respondents held a positive attitude toward the accuracy and development of DGTs. This survey can point out the direction for the improvement of DGTs, and provide a reference for the study of factors affecting implant accuracy, the establishment of a training system, and the understanding of clinicians’ current views on DGTs.

*Trial registration* This survey was approved by the Ethics Review Committee of Chenghuaxinguanghua Dental Clinic (Approval NO. CDCIRB-D-2021-201).

## Background

Dental implantation is one of the main methods for clinical treatment of dentition defects or missing. With the development of computer and digital technologies, the application of digital technologies has begun to play an important role in the field of dental implantation [1]. Digital guide template (DGT) is a surgical device that helps to achieve precise positioning through CBCT, 3D reconstruction, and CAD/CAM. It is a personalized surgical aid tool designed and manufactured for the realization of implant program [2]. Digital-guided oral implant surgery is considered to be safer and more convenient than traditional one [3], and can truly realize the concept of restoration-oriented implantation [4–6]. However, it has not been fully verified whether these new digital technologies have superior accuracy compared to the conventional techniques [7, 8]. Safety concerns make it difficult to adopt a new technology with poor accuracy. Moreover, the accuracy of surgical guide templates is particularly important due to their direct relation to the surgery. [9].

More and more related studies have been published due to the development and wide clinical application of digital-guided technologies, but they focus mostly on a certain specific issue such as the accuracy of DGTs and its influencing factors. Their findings vary and there is limited information on the views and attitudes of different clinicians toward the status quo and development of DGTs. Moreover, some clinicians still have doubts about the use and accuracy of DGTs [2, 9]. So far, a comprehensive understanding of DGTs has not been formed, and relevant questionnaire research has not been found, either. Therefore, the purpose of this survey is to investigate the application of DGTs across China, and the views and attitudes of oral health professionals toward the status quo and development of DGTs, particularly the problems related to their accuracy, so as to comprehensively understand the status quo and prospects of DGTs in the minds of their users, and help to point out the direction for relevant studies.

## Methods

### Questionnaire

Based on a large number of literature reviews and a series of visits to dental clinicians, the questionnaire was summarized by the College of Biomedical Engineering, Sichuan University. After the preliminary design of the questionnaire, seven dental implant experts and professors were invited to revise it, and then the final draft was formed. WJX (http://www.wjx.cn, an online questionnaire platform) was used for questionnaire preparation, distribution, and data recovery. There were 18 questions in the questionnaire (Table 1), and the number of answers ≥ 12 was considered valid. The contents of the questionnaire were divided into the following three parts: basic information of respondents, application of DGTs, and respondents’ views and attitudes toward the status quo and development of DGTs. Chinese oral health professionals and academic teams were invited to participate in the survey through WeChat (a popular online multimedia messaging application in China). This questionnaire was issued on December 5, 2021 and withdrawn on January 22, 2022. This study obtained approval from the Ethics Review Committee of Chenghuaxinguanghua Dental Clinic (Approval NO. CDCIRB-D-2021-201) and all methods were performed in accordance with the Declaration of Helsinki and relevant guidelines and regulations. Participants were informed that participation was anonymous and voluntary.

**Table 1:** Contents of the questionnaire

1. Basic information of respondents
(1) Where do you live? (2) What is your professional background? (3) How long have you been engaged in the oral implant industry? (4) Have you ever performed dental implantation with freehand (FH) in clinical practice? (5) Have you ever performed dental implantation with DGTs in clinical practice?
2. Application of DGTs
(1) What are the restrictions of DGTs when you use them for implantation? (2) What are the supported forms of DGTs when you use them for implantation? (3) What are the fabrication types of DGTs when you use them for implantation?
3. Views and attitudes toward the status quo and development of DGTs (1) What is your opinion on the accuracy of DGT implantation and FH implantation? (2) Do you think the accuracy (namely the deviation between preoperative design and postoperative implant placement) of DGT-guided dental implantation meets clinical requirements? (3) Do you think the influence of surgical experience on implant accuracy can be eliminated by using DGTs? (4) The preoperative design and the production of DGTs will bring extra time and economic costs. Do you think it is still worthwhile? (5) What do you think are the important factors affecting the accuracy of DGT-guided implantation? (6) Under what conditions do you think DGTs will be considered in clinical practice? (7) Will you recommend other clinicians to use DGTs for implantation? (8) Which kinds of training do you think medical staff need to participate before performing DGT-guided implant surgery? (9) Which kinds of training in the above question are more important in your opinion? (10) What is your opinion about the development status and future trend of DGT-guided implantation?

### Statistical analyses

SPSS 23.0 software was used for data collation and analysis, and Graphpad Prism 9.3 software was used for graph rendering. The basic information of respondents, the application of DGTs, and the status quo and development of DGTs were statistically described. Chi-square test was used to preliminarily evaluate the correlation between the three related factors (professional backgrounds, working years, and practice areas) and the application of DGTs as well as the views and attitudes toward DGTs.

## Results

A total of 276 questionnaires were collected, of which 273 were valid.

### Basic information of respondents

The basic information of respondents is shown in Table 2. The respondents from Sichuan Province accounted for the largest proportion, which was 29.3%. Besides, 74.7% of the respondents were dental clinical (implant) staff, 30.4% had been practicing for more than 10 years, 82.8% had performed oral implant surgery with freehand (FH), and 70.7% had used DGTs.

**Table 2:** Basic information of respondents

Basic information	Number	Percentage (%)
Practice areas		
Sichuan	80	29.3
Zhejiang	46	16.9
Chongqing	26	9.5
Guangdong	24	8.8
Guangxi	23	8.4
Shandong	15	5.5
Tianjin	14	5.1
Beijing	10	3.7
Anhui	8	2.9
Shanghai	7	2.6
Other	20	7.3
Total	273	100
Professional backgrounds (multiple choices, the total percentage may exceed 100%)		
Dental clinical (implant) staff	204	74.7
Dental clinical (non-implant) staff	65	23.8
Dental researcher	16	5.9
Dental technician	7	2.6
Dental instrument manufacturer	4	1.5
Dental digital promotion and sales staff	3	1.1
Non-dental professionals	1	0.4
Other	3	1.1
Working years		
More than 10 years	83	30.4
5–10 years	69	25.3
2–5 years	59	21.6
0–2 years	40	14.6
Not engaged in	22	8.1
Total	273	100
Whether FH implantation has been performed clinically		
Yes	226	82.8
No	46	16.8
Unfilled	1	0.4
Total	273	100
Whether DGT-guided implantation has been performed clinically		
Yes	193	70.7
No	79	28.9
Unfilled	1	0.4
Total	273	100

**Fig. 1:**
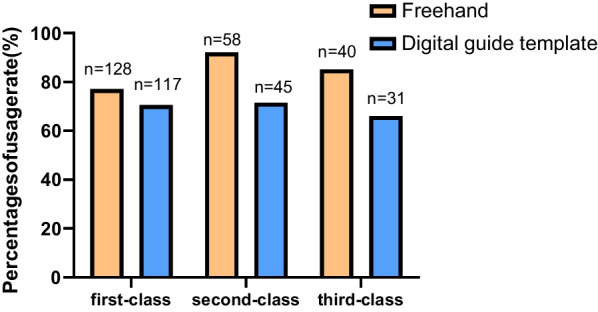
The usage rate of FH and DGTs in cities of different classes

According to the “Ranking of Cities’ Business Attractiveness 2021” [10], the respondents’ regions were divided into three classes, namely Tier 1 and New Tier 1 cities were first-class, Tier 2 were second-class, and Tier 3 and below were third-class. There were 164, 63, and 46 respondents in the first-, second-, and third-class cities, respectively. 78.0% of the respondents had used FH implantation and 71.3% had used DGTs in the first-class cities, 92.1% with FH and 71.4% with DGTs in the second-class, and 87.0% with FH and 67.4% with DGTs in the third-class, as shown in Fig. 1.

**Fig. 2:**
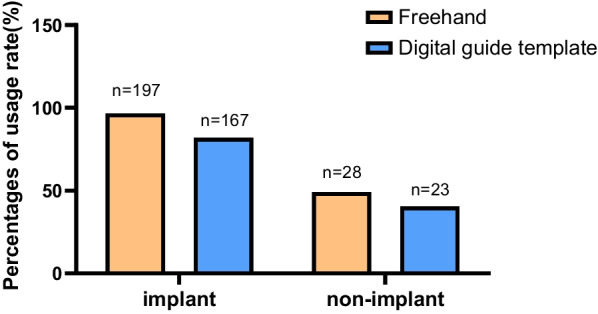
The usage rate of FH and DGTs in clinical staff

86.5% (225/260) of the dental clinical staff had used FH implantation, 96.6% (197/204) of the implant staff and 50.0% (28/56) of the non-implant staff. 73.1% (190/260) of the dental clinical staff had used DGTs, 81.9% (167/204) of the implant staff and 41.1% (23/56) of the non-implant staff, as shown in Fig. 2.

**Fig. 3:**
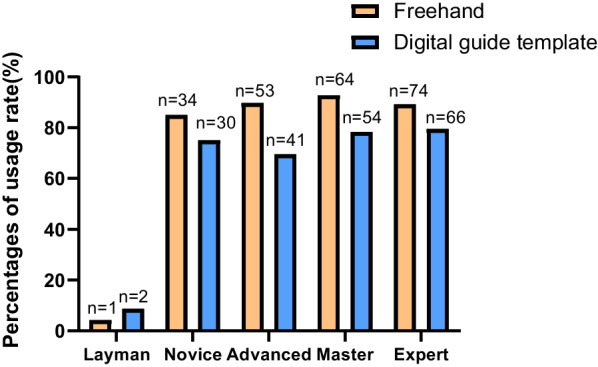
The usage rate of FH and DGTs in different levels of the respondents

The respondents were divided into Layman (not engaged in), Novice (0–2 years), Advanced (2–5 years), Master (5–10 years), and Expert (more than 10 years) according to their working years. The situations of the respondents using FH and DGTs in different levels are shown in Table 3; Fig. 3.

**Table 3:** The usage rate of FH and DGTs in different levels of the respondents

Levels of the respondents	Have you ever used FH		Have you ever used DGTs	
	Yes	No	Yes	No
	n = 226 (%)	n = 46 (%)	n = 193 (%)	n = 79 (%)
Layman	1 (4.5)	21 (95.5)	2 (9.1)	20 (90.9)
Novice	34 (85.0)	6 (15.0)	30 (75.0)	10 (25.0)
Advanced	53 (89.8)	6 (10.2)	41 (69.5)	18 (30.5)
Master	64 (92.8)	4 (5.8)	54 (78.3)	14 (20.3)
Expert	74 (89.2)	9 (10.8)	66 (79.5)	17 (20.5)

### Application of DGTs

**Fig. 4:**
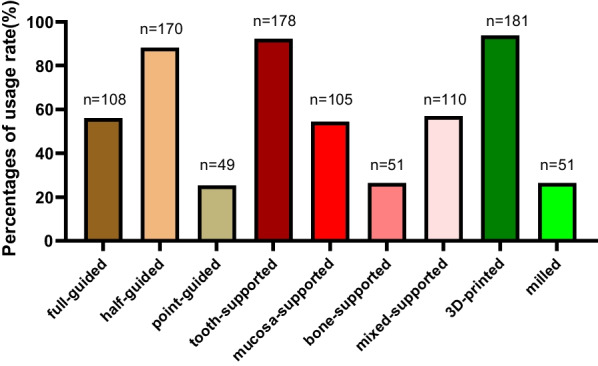
The usage rate of different types of DGTs

193 respondents had used DGTs, among which 88.1% had used half-guided templates, 56.0% full-guided templates, and 25.4% point-guided templates; 92.2% tooth-supported templates, 57.0% mixed-supported templates, 54.4% mucosa-supported templates, and 26.4% bone-supported templates; 93.8% 3D-printed guide templates, and 26.4% milled guide templates, as shown in Fig. 4.

The chi-square test revealed that working years were significantly correlated with half-guided templates among the restricted type, and tooth-supported templates as well as mucosa-supported templates among the supported type (*P* < 0.05), as shown in Table 4.

**Table 4:** The correlation between working years and application of DGTs analyzed by the chi-square test

Application of DGTs		Layman	Novice	Advanced	Master	Expert	*P*
Restricted type							
Full-guided n = 108 (%)		1 (0.9)	16 (14.8)	25 (23.1)	22 (20.4)	44 (40.7)	0.070
Half-guided n = 170 (%)		2 (1.2)	21 (12.4)	33 (19.4)	50 (29.4)	64 (37.6)	0.001**
Point-guided n = 49 (%)		1 (2.0)	5 (10.2)	9 (18.4)	16 (32.7)	18 (36.7)	0.603
Supported type							
Tooth-supported n = 178 (%)		2 (1.1)	27 (15.2)	41 (23.0)	45 (25.3)	63 (35.4)	0.030*
Mucosa-supported n = 105 (%)		2 (1.9)	9 (8.6)	20 (19.0)	30 (28.6)	44 (41.9)	0.009**
Bone-supported n = 51 (%)		0 (0.0)	6 (11.8)	11 (21.6)	12 (23.5)	22 (43.1)	0.483
Mixed-supported n = 110 (%)		1 (0.9)	13 (11.8)	22 (20.0)	31 (28.2)	43 (39.1)	0.366
Manufacturing type							
3D-printed n = 181 (%)		2 (1.1)	26 (14.4)	39 (21.5)	49 (27.1)	65 (35.9)	0.183
Milled n = 51 (%)		0 (0.0)	7 (13.7)	13 (25.5)	10 (19.6)	21 (41.2)	0.382

* *P* < 0.05, **P < 0.01

### Views and attitudes on the status quo and development of DGTs

**Fig. 5:**
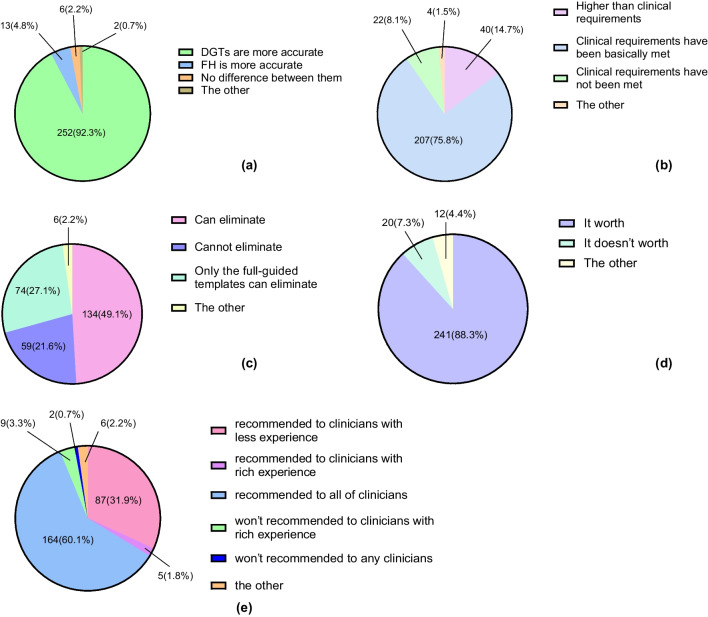
Pie charts of respondents’ attitudes on DGTs. **a** comparison of accuracy between DGTs and FH. **b** DGTs’ clinical requirements of accuracy. **c** Whether the influence of the surgical experience can be eliminated by using DGTs. **d** DGTs’ costs. **e** Clinicians’ recommendations.

Among the 273 questionnaires, the respondents’ views on the comparison of the accuracy between DGTs and FH, whether the accuracy of DGTs meets clinical requirements, whether DGTs can eliminate the influence of surgical experience on implant accuracy, whether the extra time and economic costs of using DGTs are worthwhile, and whether it is recommended to use DGTs are shown in Fig. 5. The chi-square test indicated that the respondents’ professional backgrounds and working years had significant differences in their views and attitudes toward DGTs, as shown in Table 5. More than 90% of the clinical staff thought that DGTs were more accurate than FH, while a small number of Masters and Experts with more than 5 years of working experience considered that FH was more accurate. Those who believed that the accuracy of DGT-guided implantation had not met the clinical requirements were mostly clinical staff. Nearly 90% of the respondents argued that the extra time and economic costs of using DGTs were worthwhile, especially for Novices.

**Fig. 6:**
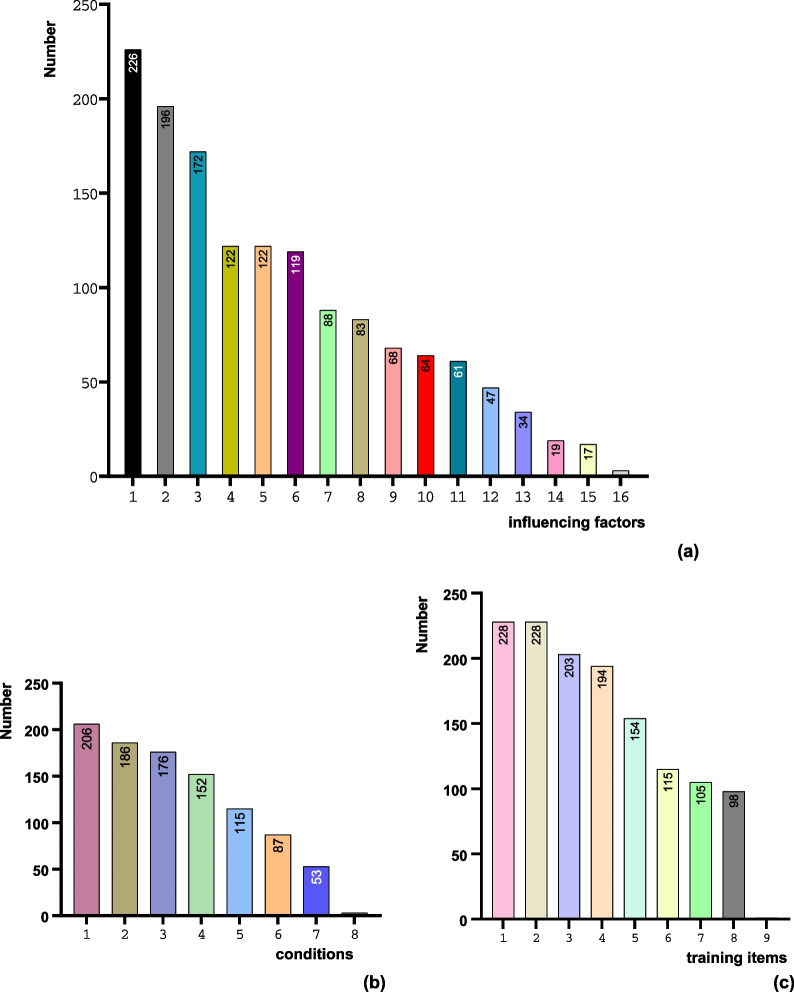
Bar graphs of respondents’ views on DGTs. **a** Influencing factors of implant accuracy. 1: the accuracy of CBCT, IOS, and other devices; 2: data collection and registration; 3: implant systems and guide design software; 4: alveolar bone morphology, cortical thickness, and bone surface gradient in the operative area; 5: the positioning and stability of DGTs; 6: the surgical areas where implants were placed; 7: the supported type of DGTs; 8: clinicians’ surgical experience; 9: clinicians’ participation in guide design; 10: preoperative design without correction; 11: BMD of operation regions; 12: the restricted type of DGTs; 13: the guide surgical tools; 14: the implants’ shape, length, diameter, etc.; 15: the manufacturing type of DGTs; 16: the other. **b** Conditions for using DGTs. 1: patients with complex anatomical structure, vascular and nerve distribution; 2: patients with many missing teeth; 3: patients who need to be guided because of restoration and aesthetic; 4: patients who need minimally invasive and flapless surgery; 5: clinicians who are inexperienced; 6: patients who need to reduce the operation time; 7: DGT is always a priority in any situation; 8: the other. **c** Training items. 1: the use of implant systems and guide design software; 2: data collection and registration of CBCT, IOS, and other devices; 3: the use and operation of DGTs and guidance tools; 4: the development of implant protocols; 5: intraoperative emergency management; 6: DGTs’ making, cleaning, disinfection; 7: methods and skills of communicating with patients; 8: FH implantation; 9: the other.

The respondents’ views on the important factors affecting the accuracy of DGT-guided implantation, the clinical considerations for the use of DGTs, and the training of clinical staff are all shown in Fig. 6.

**Table 5:** The related factors and attitudes of clinicians toward DGTs analyzed by the chi-square test

Attitudes toward DGTs	Professional backgrounds of clinicians				Working years					
	Clinical implant	Clinical non-implant	Non-clinical	*P*	Layman	Novice	Advanced	Master	Expert	*P*
	n = 204 (%)	n = 56 (%)	n = 13 (%)		n = 22 (%)	n = 40 (%)	n = 59 (%)	n = 69 (%)	n = 83 (%)	
Views on the accuracy of DGTs and FH				0.016*						0.005**
DGTs are more accurate	189 (92.6)	52 (92.9)	11 (84.6)		18 (81.8)	38 (95.0)	57 (96.6)	64 (92.8)	75 (90.4)	
FH is more accurate	12 (5.9)	1 (1.8)	0 (0.0)		1 (4.5)	0 (0.0)	0 (0.0)	5 (7.2)	7 (8.4)	
There’s not much difference between them	2 (1.0)	2 (3.6)	2 (15.4)		3 (13.6)	1 (2.5)	2 (3.4)	0 (0.0)	0 (0.0)	
Whether the accuracy of DGT-guided implantation meets clinical requirements?				0.047*						0.048*
Higher than clinical requirements	29 (14.2)	8 (14.3)	3 (23.1)		5 (22.7)	9 (22.5)	5 (8.5)	11 (15.9)	10 (12.0)	
Clinical requirements have been basically met	162 (79.4)	36 (64.3)	9 (69.2)		11 (50.0)	30 (75.0)	48 (81.4)	54 (78.3)	64 (77.1)	
Clinical requirements have not been met	11 (5.4)	10 (17.9)	1 (7.7)		6 (27.3)	1 (2.5)	5 (8.5)	3 (4.3)	7 (8.4)	
Can the use of DGTs eliminate the influence of surgical experience on accuracy				0.217						0.980
Yes	108 (52.9)	23 (41.1)	3 (23.1)		9 (40.9)	20 (50.0)	30 (50.8)	33 (47.8)	42 (50.6)	
No	43 (21.1)	12 (21.4)	4 (30.8)		5 (22.7)	8 (20.0)	10 (16.9)	15 (21.7)	21 (25.3)	
Only the full-guided templates can do	48 (23.5)	20 (35.7)	6 (46.2)		8 (36.4)	11 (27.5)	18 (30.5)	19 (27.5)	18 (21.7)	
Are the extra time and economic costs of using DGTs worthwhile?				0.390						0.019*
Yes	182 (89.2)	49 (87.5)	10 (76.9)		15 (68.2)	39 (97.5)	49 (83.1)	65 (94.2)	73 (88.0)	
No	13 (6.4)	6 (10.7)	1 (7.7)		5 (22.7)	0 (0.0)	7 (11.9)	1 (1.4)	7 (8.4)	

* *P* < 0.05, **P < 0.01

## Discussion

### Basic information of respondents

Chinese oral health professionals were invited to fill in the questionnaires, and a relatively ideal response effect was achieved in this survey. However, as the questionnaire was distributed to target audience groups or individuals extensively or separately through WeChat, the total number of people who have consulted the questionnaire cannot be counted, so the response rate cannot be obtained, which is the deficiency of this questionnaire distribution. However, the effective rate of the 276 questionnaires collected reached 98.91%, and only 3 questionnaires were judged to be invalid (the number of answers were: 6, 1, 2). Among the 273 valid questionnaires, a total of 260 (95.2%) respondents were dental clinical workers, 204 (74.7%) were dental clinical implant workers, 152 (55.7%) had been engaged in the implant industry for more than 5 years, and 184 (67.4%) had used both FH and DGTs for implant surgery. Therefore, the results of this survey are of great reference value in terms of professionalism.

According to the survey, the usage rate of FH (82.8%) was higher than that of DGTs (70.7%), and there was an obvious regional difference. In the first-class cities, the difference was small. In the second and third-class cities, the usage rate of DGTs was significantly lower than that of FH. This proves that DGTs are popularized from strong economic areas to relatively weak ones, and there is still a lot of room for improvement in the popularization.

### Application of DGTs

#### The restricted type

The half-guided type is more accurate than the point-guided one, and also it is more convenient and more flexible than the full-guided one, so it is more common in clinical application in China. This survey confirmed that the usage rate of the half-guided templates was the highest, accounting for 88.1% of the respondents who had used DGTs, and it increased gradually with working years. In addition, this survey showed that the usage rate of the point-guided templates was the lowest, and most of them had worked for more than 5 years. The usage rate of the full-guided templates only accounted for 56.0% of those who had used DGTs, and 40% of them were experts with more than 10 years of working experience, while other levels only accounted for 10-20%. However, it has been reported in lots of literature [11–14] that the full-guided type has the highest accuracy. Younes [15] advocated that the full-guided surgery “should be considered the gold standard approach”. Also, there are studies [13, 16] (All belong to in vitro model experiments) concluding that there is no difference in the accuracy of the full-guided type achieved between inexperienced and experienced clinicians. These results coincide with the important purpose of using guide templates-to lower the threshold of experience required by clinicians in implantation through the assistance of the guide. In this survey, 76.2% of the respondents believed that the full-guided type could eliminate the influence of surgical experience on implant accuracy.

The full-guided type has the highest accuracy, while its usage rate is low in China, which may be related to the fact that it cannot be adjusted during the operation. This feature means that the full-guided type needs to meet a high standard from the initial design to the completion of manufacturing and then to the placement of the guide; otherwise, its effect will be greatly reduced. Also, this requires the staff at all levels to have more professional skills, which is still a long way to go in China where systematic training is lacking. In addition, the cost of the full-guided templates is high, and the operation steps are complicated, making it difficult to improve its usage rate. Therefore, it is both an expectation and a challenge for the full-guided templates to be popularized in China.

#### The supported type

It is concluded from a systematic review and meta-analysis [17] that tooth-supported type is more accurate than bone-supported and mucosa-supported ones. Tahmaseb et al. [18] also reported that tooth- and mucosa-supported guides are more accurate than bone-supported ones. There is almost a consensus that the accuracy of the tooth-supported guide is higher. Thus, it has a relatively significant usage rate. In this survey, the usage rate of the tooth-supported guide templates accounted for 92.2% of the respondents who had used DGTs. The chi-square test showed that there was a significant difference between the supported type and clinicians’ working years (*P* < 0.05), and the usage rate of mucosa-, mixed- and bone-supported guide templates increased gradually with the working years.

#### The manufacturing type

According to this survey, the usage rate of 3D-printed guide templates is much higher than that of milled guide templates. Additive manufacturing is utilized more than subtractive manufacturing, which, on the one hand, reflects the rapid development of 3D printing technology, whose production accuracy can meet clinical requirements, and on the other hand demonstrates the fact that subtractive technology consumes more raw materials and increases the production cost. But studies vary on their accuracy. In vitro studies by Henprasert et al. [19] and Chai et al. [20] showed that guide templates made with additive or subtractive technology do not affect implant accuracy. In contrast, Abduo et al. [21] proved in another study that more accurate results can be obtained by milled guide templates.

### Views and attitudes toward the status quo and development of DGTs

#### Views on the accuracy of DGTs

According to the results of this survey, 92.3% of the respondents believed that the accuracy of DGTs was higher than that of FH; 90.5% believed that the accuracy of DGTs was higher than or basically met the clinical requirements; and 88.3% believed that it was worth paying extra time and economic costs for DGTs. Interestingly, the few (about 5-8%) who held a negative attitude toward the above problems were mostly Experts with more than 10 years of surgical experience, suggesting to some extent that the use of DGTs is of limited help to clinicians with rich surgical experience, while it plays a better role in helping and guiding clinicians with less experience. Many studies [1, 11, 15, 22, 23] proved that using DGTs is more accurate than using FH. Murat [24] pointed out that the implant moves along the path of least resistance in FH implantation, which can easily lead to large deviations, especially for patients with relatively low bone mineral density (BMD). There is also a study on the extra time cost of DGTs: Martelli et al. [25] pointed out that time saved is subjective and depends on the perspective used to assess or appreciate the time saved. In monetary terms, for example, 10 min saved in an operating room can potentially have the same value as 1 h of work on the object design or its production. In this survey, 12 respondents made written explanations on this aspect, and their views on the cost depended on specific cases. For the cases with complicated implants or with a large number of missing teeth, the cost would be worthwhile, while for the cases with simple implants, it would be considered otherwise.

#### Views on relevant factors affecting the accuracy of DGTs

The accuracy of DGT-guided implantation is the most concerning issue in clinical practice, and many steps can lead to the deviations of implant placement and design. At present, different influencing factors have been discussed in numerous studies. This survey collected and summarized a relatively comprehensive list of possible influencing factors (15 items in total), and they were in order of the selection rate from high to low as follows: the accuracy of CBCT, intraoral scanning (IOS), and other devices (82.8%); data collection and registration (71.8%); implant systems and guide design software (63.0%); alveolar bone morphology, cortical thickness and bone surface gradient in the operative area (44.7%); the positioning and stability of DGTs (44.7%); the surgical areas where implants were placed (43.6%); the supported forms of DGTs (32.2%); clinicians’ surgical experience (30.4%); clinicians’ participation in guide design (24.9%); preoperative design without correction (23.4%); and BMD of operation regions (22.3%), etc.

It can be seen that the first three are limited by the development of hardware and software, data processing, and other technologies, indicating that most respondents still hold a skeptical attitude toward the accuracy of current digital technologies. The accuracy of CBCT measurement needs to be improved, and many scholars have conducted research in this area. There is a study [2] pointing out that the scanning layer thickness of CBCT is 0.2 ~ 0.4 mm, which determines the accuracy of CBCT and affects the design of subsequent schemes. Komuro et al. [26] examined dimensional reproducibility and shrinkage rates measured by the same subject using a model scanner, IOS, and CBCT, respectively. It is found that the measured values of CBCT are significantly lower than those of the model scanner and IOS (*P* < 0.001). Other scholars [27] studied the influence of registration conditions of optical scanning images and radiation images on the accuracy and found that the accuracy based on full-surface registration is higher than that based on the local matching. In the study of the implant systems and guide design software, Ashtiani et al. [28] reviewed the deviations of 6 kinds of guide design software, among which 3Shape shows smaller mean angle deviation, coronal deviation, and apical deviation. Vasak et al. [29] reported that NobelGuide systems get similar or less spatial and angular deviation than other software. Murat [24] studied the accuracy of StentCad Beyond guidance system and found that it was similar to the average depth deviation (0.6 ± 0.4 mm), average shoulder deviation (1.5 ± 0.8 mm), and average angle deviation (7.9 °±5 °) produced by Simplant/SurgiGuide.

There is also some literature on the effects of surgical area, BMD, alveolar bone morphology, cortical thickness, and bone surface gradient. In molar area, Lin et al. [30] pointed out that the guide template usually with only one end supported on the remaining teeth. Although the tooth-supported guide was considered to be capable to achieve more accuracy, this distal free-end situation of the guide could result in insufficient stability, and thus reduced accuracy. López et al. [31] noted that micro-movements of the guide template might arise during drilling, and the use of the unilaterally supported guide template might lead to large implant deviations due to the tilt and bending of the guide template. EI Kholy et al. [32] reported in an in vitro model experiment, that implants placed distal had significantly greater shoulder and root deviations compared with implants placed in bilateral retainers. Tang et al. [33] studied that the quadrant factor affected buccolingual direction deviations at the apical point. Zhou et al. [34] pointed out that the implant placement in the mandible had a smaller angle deviation than that in the maxilla, which could be explained from the perspective of bone anatomy and BMD. The structure of the mandible was a straight bow, while the shape of the maxilla was a round curve, which limited the control of angle, what’s more, the density of the mandible was higher. Vinci et al. [35] concluded in a study of the accuracy of the mucosa-guided in edentulous patients, that errors in the mandible were greater than those in the maxilla, and errors in the posterior region were greater than those in the anterior teeth of the maxilla and mandible. Some scholars also pointed out that the tooth position in the surgical area had no significant effect on the accuracy [36]. Kivovics et al. [37] resulted in a weak and statistically significant negative correlation between BMD and angular deviation, the higher the BMD of the planting regions, the higher the accuracy. A possible interpretation of this result might be that it was more difficult to deviate from the path of the pilot drill in denser bone.

The three factors, including the placement and stability of DGTs, the clinicians’ participation in the design of DGTs, and the preoperative design without correction, are summarized according to the experience of clinicians. However, currently there are few reports on these three aspects. First, the placement and stability of DGTs involve the degree of fitting between DGTs and patients’ surgical area, the imaging accuracy, the fitting accuracy of design software, the design and production of DGTs, the specific situation of patients, the surgical experience, etc., and it is necessary to set up a comprehensive consideration of multiple variables to do the research. Lim et al. [38] significantly improved the accuracy of the guide template by considering the seal and offset of the occlusal groove in the design and manufacture. Second, clinician’s participation in the design of DGTs reflects whether the design has sufficient clinical expertise. But there is a lack of comprehensive training system in China at present. Some clinicians have poor software operation skills and can only leave the design to technicians, while they possess less clinical operation experience, which inevitably leads to errors in the design. To reconcile this contradiction, it is necessary to increase the participation of clinicians in the design. Besides, the subsequent training system for clinicians and technicians should be improved effectively. Third, in terms of the preoperative design without correction, a large number of retrospective studies and clinicians’ experience summaries should be required to find the causes of the postoperative deviations. The principle of beginning with the end in mind should be applied to correct the deviations in the design of DGTs, in order to achieve the purpose of the actual postoperative implantation position closer to the ideal position. Moreover, a highly professional and perfect cooperation between technicians and clinicians is also needed, and only with the support of a large number of experimental data can the degree of correction during the design be quantified.

The supported forms of DGTs have been discussed in the previous part, and the following part will discuss clinicians’ surgical experience. The above survey results can provide more targeted and directional reference value for subsequent research on the factors influencing the accuracy of DGT-guided implantation.

#### Influence of surgical experience on the accuracy of DGT-guided implantation

The influence of clinicians’ surgical experience on the accuracy of DGT-guided implantation is a research hotspot because it almost determines whether novice clinicians can be free to use DGTs for implantation. Lin et al. [39] showed that when the tooth-supported guide template was used in a well-controlled environment, the surgical experience was not the key factor affecting the implant accuracy. Some scholars [37] studied the influence of surgical experience on the accuracy of the half-guided and the mucosa-supported guide templates and reached the same conclusion. Another study [40] showed that the depth deviation of the implant had the greatest impact on the surgical experience level (in vitro model experiment). In this survey, 49.1% of the respondents thought that the influence of surgical experience on the accuracy of implantation could be eliminated by DGTs, 42.1% of the respondents thought that DGTs should be considered when clinicians were inexperienced, and 30.4% of the respondents thought that surgical experience would affect the accuracy of DGT-guided implantation. Moreover, 91.9% of the respondents would recommend DGTs to clinicians with little surgical experience, and 35.9% considered that medical staff should be trained in FH implantation before performing DGT-guided implantation. At present, most studies on the influence of surgical experience on implant accuracy are in vitro model experiments. Based on the current level of digital technology and the accuracy of DGTs, both the strict requirements of professionalism in the design and manufacture of the full-guided templates and the flexible operation of the half- and point-guided templates during surgery require clinicians to have extensive surgical experience. To completely achieve the goal of obtaining high precision of implantation without the experience level of clinicians and directly relying on DGTs, it still needs the development and promotion of all aspects of relevant technologies.

The written views of 78 respondents on DGTs were also collected in the questionnaires. Most of them believed that using DGTs was a general trend, and it would have a better development in the future, but it was necessary to reduce the cost, and improve the accuracy and patients’ understanding of DGTs to facilitate the promotion of DGTs. Many respondents hoped that clinicians should be involved in the design of DGTs and relevant systematic training should be provided. The results of this survey found the five training items with the highest selection rate as follows: training on the use of implant systems and guide design software (82.6%); training on data collection and registration of CBCT, IOS and other devices (82.6%); training on the use and operation of DGTs and guidance tools (73.6%); training on the development of implant protocols (70.3%); and training on intraoperative emergency management (55.9%). Some respondents proposed that novice clinicians should not rely too much on DGTs and they should learn FH implantation first, the full-guided templates should be promoted, the development and application of domestic implantation systems and software should be accelerated, and the improvement of DGTs itself should be based on clinical practice such as the lateral opening of the posterior dental area to reduce the impact of mouth opening. Some respondents believed that restoration- and aesthetic-oriented implants required DGTs more, but DGTs were not suitable for patients with the irregular shape of alveolar bone, bone grafting, and intraoperative adjustment. The results of this survey indicated that DGTs might be considered in the following clinical situations: patients with complex anatomical structures, vascular and nerve distributions (74.6%); patients with many missing teeth (67.4%); patients who need to be guided because of restoration and aesthetic (63.8%); patients need minimally invasive and flapless surgery (55.1%); clinicians who are inexperienced (41.7%); and patients who need to reduce the operation time (31.5%). There were 19.2% of the respondents who would give priority to DGTs no matter what the situation was. Some respondents raised the following concerns: the popularity of DGTs would confuse the industry to a certain extent, clinicians with poor skills dared to perform implant surgery, so industry standards needed to be developed; CBCT-based DGTs could not solve soft tissue problems such as insufficient attached gingival; bone burn could be easily caused without the cooling system; and digital promotion would reduce the value of experienced clinicians. Some others predict that digital navigation would eventually replace DGTs.

## Conclusion

This survey preliminarily revealed the views of Chinese oral health professionals on the application, status quo, and development of DGTs. Through the classifications of the respondents by regions, professional backgrounds, and working years, it was found that there were significant differences in the application and views of DGTs among different groups. The results of this survey can point out the direction of the improvement of DGTs, the study of factors affecting implant accuracy, the establishment of a training system, and the understanding of clinicians’ current views on DGTs. However, due to the limited invitation of clinicians and the small number of respondents from different regions, professional backgrounds, and working years, it is difficult to form a relatively complete evaluation, which is the deficiency of this survey.

## Data Availability

The datasets generated and/or analysed during the current study are not publicly available due to personal privacy, but are available from the corresponding author on reasonable request.

## Abbreviations

DGT/DGTs: Digital guide template/digital guide templates
CBCT: Cone beam computed tomography
FH: Freehand
BMD: Bone mineral density
IOS: Intraoral scanning.
